# Predictive value of a nomogram for hepatocellular carcinoma with brain metastasis at initial diagnosis: A population-based study

**DOI:** 10.1371/journal.pone.0209293

**Published:** 2019-01-02

**Authors:** Qi-Feng Chen, Tao Huang, Lujun Shen, Wang Li

**Affiliations:** 1 State Key Laboratory of Oncology in South China, Collaborative Innovation Center for Cancer Medicine, Sun Yat-sen University Cancer Center, Guangzhou, Guangdong, P.R. China; 2 Department of Medical Imaging and Interventional Radiology, Sun Yat-sen University Cancer Center, Guangzhou, Guangdong, P.R. China; University of North Carolina at Chapel Hill School of Medicine, UNITED STATES

## Abstract

**Background:**

Population-based estimates of the incidence and prognosis of brain metastases at diagnosis of hepatocellular carcinoma (HCC) are lacking. The aim of this study was to characterize the incidence proportion and survival of newly diagnosed hepatocellular carcinoma with brain metastases (HCCBM).

**Materials and methods:**

Data from Surveillance, Epidemiology, and End Results (SEER) program between 2010 and 2014 was evaluated. Patients with HCCBM were included. Multivariable logistic and Cox regression were performed to identify predictors of the presence of brain metastases at diagnosis and prognostic factors of overall survival (OS). We also built a nomogram based on Cox model to predict prognosis for HCCBM patients.

**Results:**

We identified 97 patients with brain metastases at the time of diagnosis of HCC, representing 0.33% of the entire cohort. Logistic regression showed patients with bone or lung metastases had greater odds of having brain metastases at diagnosis. Median OS for HCCBM was 2.40 months. Cox regression revealed unmarried and bone metastases patients suffered significantly shorter survival time. A nomogram was developed with internal validation concordance index of 0.639.

**Conclusions:**

This study provided population-based estimates of the incidence and prognosis for HCCBM patients. The nomogram could be a convenient individualized predictive tool for prognosis.

## Introduction

Hepatocellular carcinoma (HCC) represents the most common type of liver cancer, which is the third leading cause of cancer deaths worldwide [1]. Although HCC with extrahepatic metastasis is not uncommon (18.4% in 7681 patients when HCC were newly diagnosed) [2], intracranial metastases is relatively rare. Suffering hepatocellular carcinoma brain metastases (HCCBM) dramatically worsen patients’ prognosis. The clinical course of these patients remains unclear [3], with a median survival that has been described as between 1 months to 1.7 months [4, 5]. Therefore, Brain metastases represent an important cause of morbidity and mortality [4].

However, only a few related studies have been reported to date, and a large part of them were case reports [6–16]. Moreover, previously published data focused primarily on relatively small sample size patients treated at medical centers. Epidemiology and clinical predictors of outcome, on a population level in HCCBM, have not been well characterized. Lacking data about patient characteristics and prognostic factors in this unique group of patients makes the prognostic assessment and management very challenging. Population-level estimates for probability and prognosis among patients with newly diagnosed HCC and brain metastases are therefore warranted for clinical decision.

The purpose of this study was to use the Surveillance, Epidemiology, and End Results (SEER) database to analyze the incidence of HCCBM and possible prognostic factors in the survival of patients who present with brain metastases in newly diagnosed HCC. Furthermore, the Cox regression models were visualized in nomogram, which has been applied to predict cancer patients’ prognosis [17]. We also built a user-friendly nomogram to evaluate the probability of survival rate.

## Materials and methods

**Fig 1:**
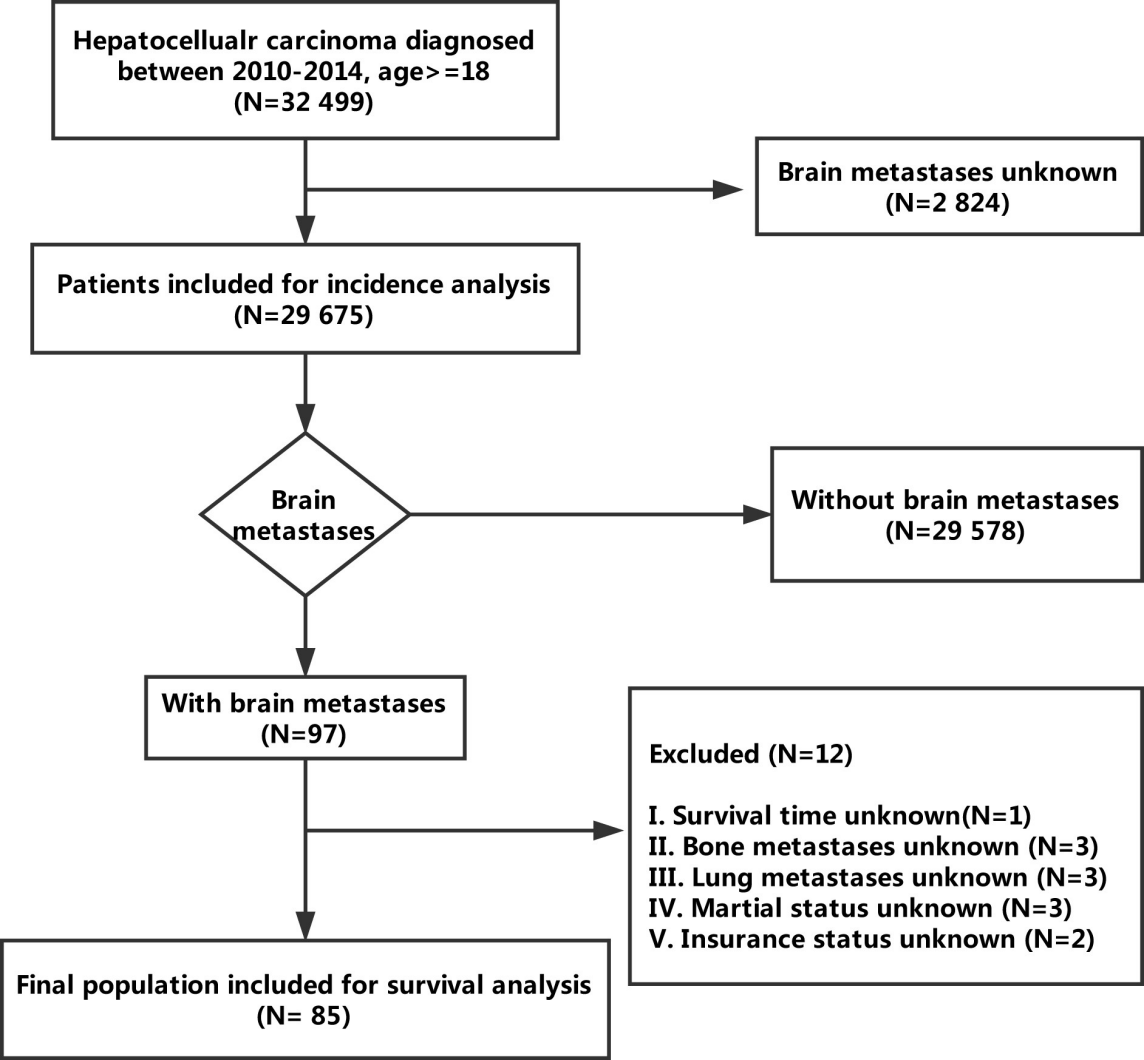
Flowchart of patient selection.

Data were obtained from the SEER program, covering approximately 30% of the US population. SEER currently collects data on cancer incidence, treatment, and survival data. We obtained information on the presence or absence of brain metastases in newly diagnosed HCC patients after obtaining permission to access research data files with the reference number 16136-Nov2016. We defined the study interval between the year 2010 and 2014 because the sites of brain metastases at initial diagnosis were only available since the year 2010. Within the SEER database, we identified 32 499 patients 18 years or older who were diagnosed as having HCC between 2010 and 2014. Patients for whom the presence or absence of brain metastases at diagnosis was unknown were excluded (n = 2 824), leaving 29 675 patients in the final cohort for incidence analysis. Of these, 97 patients were diagnosed as having brain metastases. We subsequently removed patient who had unknown factors (n = 12), leaving 85 patients eligible for survival analysis (**Fig 1**). The data released by the SEER database contained neither human subjects nor personal identifying information in this present study, so informed consent was not required. Our study had already been approved by the Ethical Committee and Institutional Review Board of Sun Yat-Sen University Cancer Center.

Variables used in the current study were determined by previous studies [2, 18], including age, sex, race, marital status, insurance status, bone metastases, lung metastases, brain metastases, vital status, and survival months. Fisher’s exact or chi-square tests for categorical variables were calculated to compare baseline characteristics. Multivariable logistic regression was used to determine whether age, sex, race, marital status, insurance status, bone metastases, and lung metastases were associated with the presence of brain metastases at diagnosis. Overall survival (OS) was calculated from diagnosis to death from any cause. The Kaplan–Meier method and log-rank test were used to evaluate OS differences. Multivariable Cox proportional hazards models were determined; hazard ratios with 95% confidence intervals (CIs) were then calculated. Furthermore, we developed a nomogram using the rms package in R project. Additionally, the discrimination and stability of this model were calculated through c-statistics and Bootstrap sample. Discrimination, calculated through concordance index (c-index, Harrell’s overall concordance statistic), was the model's ability to differentiate between patients who die from HCCBM and patients who will not. An internal validation with 1000 sets of full bootstrap samples was performed to evaluate the ability of the nomogram. Calibration plots were drawn to compare event rate observed in the population and event rate predicted by the model for groups of patients at a certain time. All tests were two-sided; *P <* 0.05 was considered significant. All analyses were conducted using R (version 3.4.1; R Foundation).

## Results

### Incidence

**Table 1** showed the number and incidence proportions of HCC patients with (or without) identified brain metastases at initial diagnosis. Among the whole population, 97 patients presented with brain metastases, reflecting 0.33% of the entire study cohort. Other characteristics were provided in **Table 1**. HCCBM had lower rates of surgery (*P* < 0.001), higher rates of bone (*P* < 0.001) and lung (*P* < 0.001) metastases, lower rates of insurance (*P* < 0.001), and were less seemly to be married status *(P* < 0.05).

**Table 1:** Patient characteristics of hepatocellular carcinoma.

Characteristics	Whole Patients (N = 29 675)	With brain metastases (N = 97)	*P*
Year of diagnosis			0.719
2010	5314 (17.91%)	18 (18.56%)	
2011	5642 (19.01%)	21 (21.65%)	
2012	6025 (20.30%)	16 (16.49%)	
2013	6270 (21.13%)	24 (24.74%)	
2014	6424 (21.65%)	18 (18.56%)	
Age	64.0 ± 10.8 (18–102)	62.0 ± 10.7 (33–85)	0.069
Sex			0.283
Male	22802 (76.84%)	79 (81.44%)	
Female	6873 (23.16%)	18 (18.56%)	
Race			0.613
White	20577 (69.34%)	71 (73.20%)	
Black	4085 (13.77%)	14 (14.43%)	
Others	4954 (16.69%)	12 (12.37%)	
Unknown	59 (0.20%)	0 (0.00%)	
Marital status			0.030
Unmarried	6497 (21.89%)	32 (32.99%)	
Married	21567 (72.68%)	61 (62.89%)	
Unknown	1611 (5.43%)	4 (4.12%)	
Insurance status			< 0.001
Uninsured	1276 (4.30%)	7 (7.22%)	
Insured	27792 (93.65%)	86 (88.66%)	
Unknown	607 (2.05%)	4 (4.12%)	
Surgery			< 0.001
Yes	22583 (76.10%)	5 (5.15%)	
No	7008 (23.62%)	92 (94.85%)	
Unknown	84 (0.28%)	0 (0.00%)	
Brain metastases			< 0.001
Yes	97 (0.33%)	97 (100.00%)	
No	29578 (99.67%)	0 (0.00%)	
Bone metastases			< 0.001
Yes	1206 (4.06%)	40 (41.24%)	
No	28417 (95.76%)	54 (55.67%)	
Unknown	52 (0.18%)	3 (3.09%)	
Lung metastases			< 0.001
Yes	1687 (5.68%)	41 (42.27%)	
No	27833 (93.79%)	53 (54.64%)	
Unknown	155 (0.52%)	3 (3.09%)	

On multivariable logistic regression (**Table 2**) among entire population, bone metastases (vs without bone metastases; odds ratio [OR], 9.841; 95% CI, 6.236–15.531; *P* < 0.001) and unknown bone metastases (vs without bone metastases; OR, 13.467; 95% CI, 3.732–48.601; *P* < 0.001), lung metastases (vs without lung metastases; odds ratio [OR], 6.654; 95% CI, 4.221–10.492; *P* < 0.001) and unknown lung metastases (vs without lung metastases; OR, 3.812; 95% CI, 1.084–13.410; *P* < 0.001) were associated with significantly greater odds of having brain metastases at diagnosis. Other variables (age, sex, race, marital status, and insurance status) were not associated with a risk of brain metastasis at diagnosis in the multivariable model. Significant results are presented in **Table 2**.

**Table 2:** Multivariable logistic regression for the presence of brain metastases at diagnosis of hepatocellular carcinoma.

Characteristics		Odds ratio (95% CI)	*P*
Age		0.990 (0.971–1.010)	0.316
Sex	Male	Reference	0.777
	Female	0.927 (0.549–1.567)	
Race	White	Reference	0.558
	Black	0.707 (0.390–1.282)	
	Others	0.722 (0.386–1.348)	
Marital status	Unmarried	Reference	0.260
	Married	0.699 (0.439–1.114)	
	Unknown	0.558 (0.189–1.643)	
Insurance status	Uninsured	Reference	0.255
	Insured	0.839 (0.374–1.880)	
	Unknown	2.004 (0.554–7.251)	
Bone metastases	No	Reference	< 0.001
	Yes	9.841 (6.236–15.531)	
	Unknown	13.467 (3.732–48.601)	
Lung metastases	No	Reference	< 0.001
	Yes	6.654 (4.221–10.492)	
	Unknown	3.812 (1.084–13.410)	

### Survival analysis

**Fig 2:**
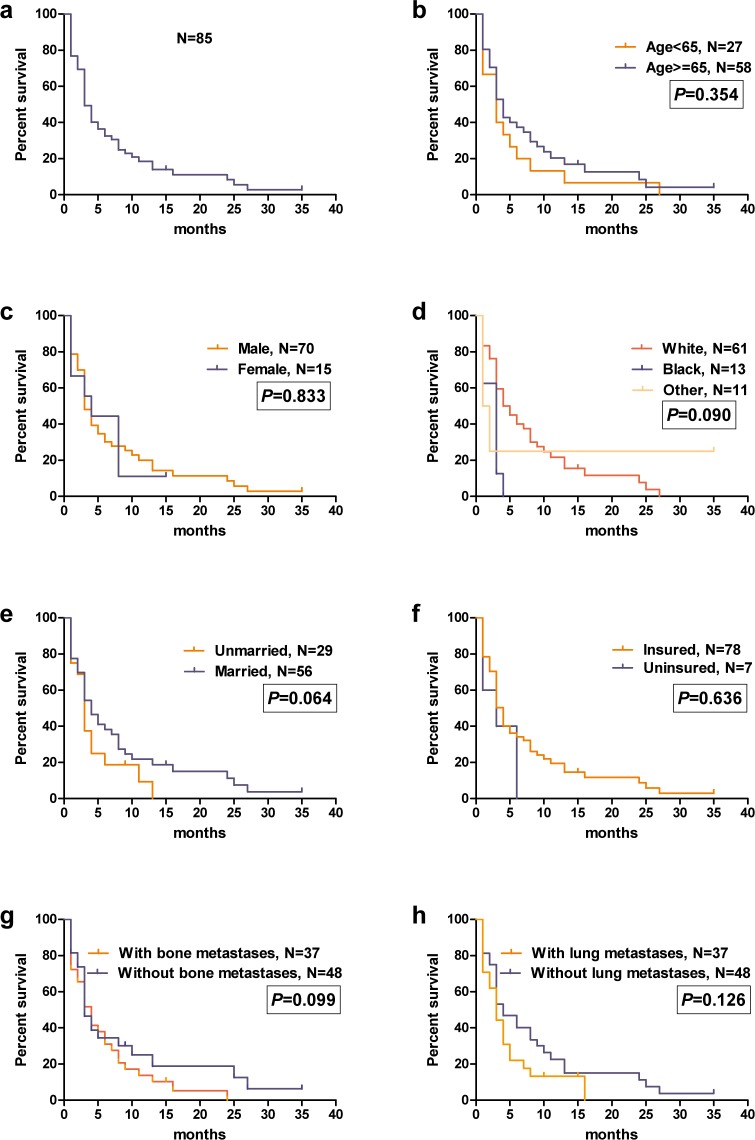
Overall survival among patient (a) and survival stratified by age (b), sex (c), race (d), marital status (e), insurance (f), bone metastases (g) and lung metastases (h).

The median follow-up period was 2 months (range, 0–35 months). Seventy seven patients suffered death. Median OS for the entire study group was 2.40 months. Survival estimates overall (**Fig 2A**) and as stratified by age (**Fig 2B**), sex (**Fig 2C**), race (**Fig 2D**), marital status (**Fig 2E**), insurance (**Fig 2F**), bone metastases (**Fig 2G**) and lung metastatic disease (**Fig 2H**) were graphically displayed in the **Fig 2**. Significant OS difference was not found between all variables, including age < 65 years (median OS: 3.00 months; 95% CI: 1.73–4.27 months) and age > = 65 years (median OS: 1.00 months; 95% CI: 0.05–1.95 months; *P* = 0.354); male (median OS: 2.00 months; 95% CI: 1.00–3.00 months) and female (median OS: 1.00 months; 95% CI: 0.00–2.93 months; *P* = 0.833); White (median OS: 3.00 months; 95% CI: 1.51–4.49 months), Black (median OS: 1.00 months; 95% CI: 0.00–2.15 months) and others (median OS: 1.00 months; 95% CI: 0.08–1.92 months; *P* = 0.090); unmarried (median OS: 1.00 months; 95% CI: 0.00–2.25 months) and married (median OS: 3.00 months; 95% CI: 1.47–4.53 months; *P* = 0.064); uninsured (median OS: 1.00 months; 95% CI: 0.00–2.28 months) and insured (median OS: 2.00 months; 95% CI: 1.00–3.00 months; *P* = 0.636); absence of bone metastases (median OS: 3.00 months; 95% CI: 2.23–3.77 months) and presence of bone metastases (median OS: 1.00 months; 95% CI: 0.00–2.32 months; *P* = 0.099); absence of lung metastases (median OS: 1.00 months; 95% CI: 0.86–1.15 months) and presence of lung metastases (median OS: 1.00 months; 95% CI: 0.90–1.10 months; *P* = 0.126).

Multivariate analysis with Cox proportional hazard model revealed that marital status (hazard ratio [HR], 0.542; 95% CI, 0.318–0.926; *P* = 0.025) and bone metastases (hazard ratio [HR], 0. 585; 95% CI, 0.357–0.962; *P* = 0. 034) were independently associated with OS. However, age, sex, race, insurance, and lung metastases were not significantly related to OS with this test (**Table 3**).

**Table 3:** Multivariable Cox regression for survival analysis among patients with brain metastases.

Characteristics		Hazard ratio (95% CI)	*P*
Age		1.010 (0.987–1.033)	0.401
Sex	Male	Reference	0.738
	Female	1.112 (0.597–2.071)	
Race	White	Reference	0.166
	Black	1.712 (0.860–3.408)	
	Others	1.217 (0.566–2.616)	
Marital status	Unmarried	Reference	0.025
	Married	0.542 (0.318–0.926)	
Insurance status	Uninsured	Reference	0.809
	Insured	0.898 (0.376–2.148)	
Bone metastases	Yes	Reference	0.034
	No	0.585 (0.357–0.962)	
Lung metastases	Yes	Reference	0.148
	No	1.421 (0.883–2.289)	

### Prognostic nomogram for OS

**Fig 3:**
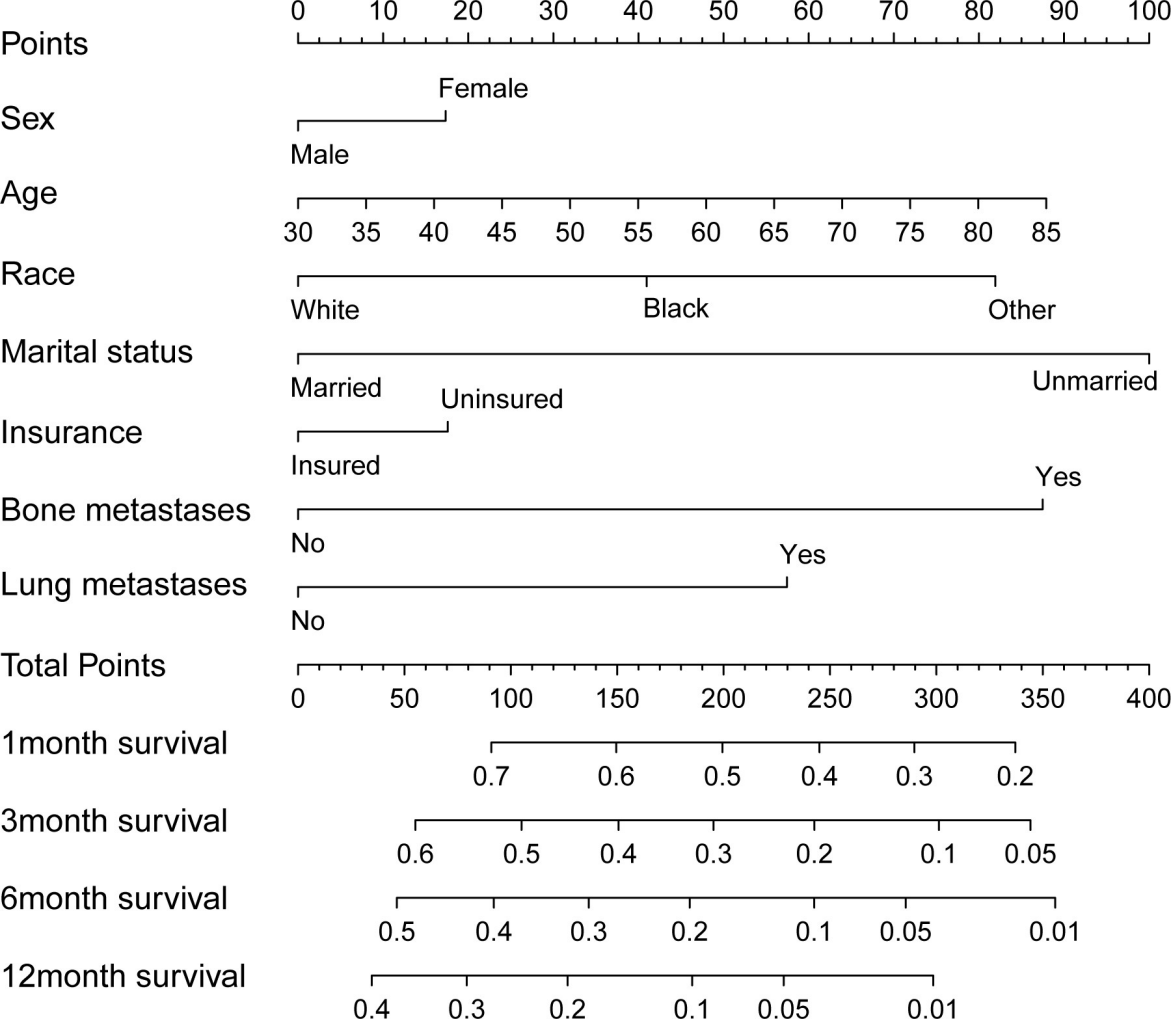
Overall survival nomogram for hepatocellular carcinoma with brain metastases.

**Fig 4:**
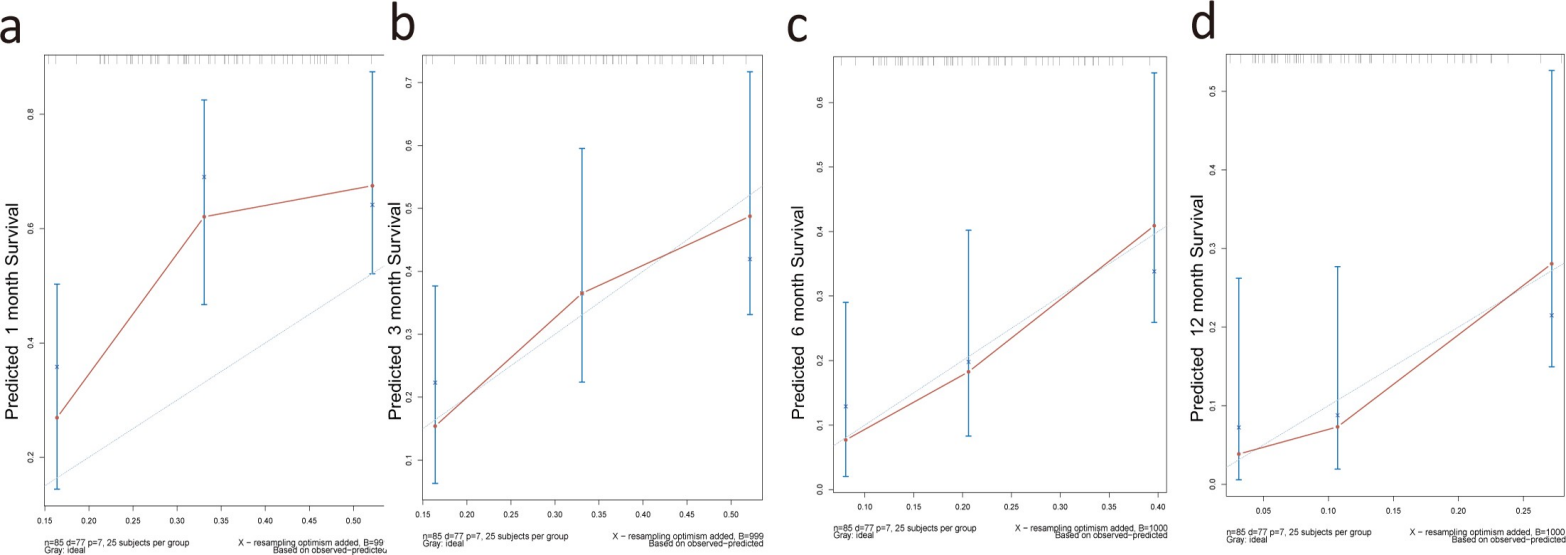
The calibration curves for predicting patient survival at 1 (a), 3 (b), 6 (c), and 12 (d) months in the study cohort.

Through the Cox regression model, we built a prognostic nomogram incorporating the above independent prognostic factors for visualization and facilitating clinical practice as shown in **Fig 3**. We used bootstrapping to internally validate the model. The C-index for OS prediction was 0.639 (95%CI, 0. 538 to 0.741). Stability and internal validation were studied over the 1000 bootstrap samples. The calibration plots, displayed in **Fig 4A**, **Fig 4B**, **Fig 4C** and **Fig 4D** for the probability of survival at 1, 3, 6, 12 month(s) after diagnosis showed a good agreement between the prediction by nomogram and actual observation.

## Discussion

There were several major findings in our study. First, we reported for the first time, to our knowledge, population-based outcomes among patients with brain metastases on initial HCC presentation. In our cohort with initial diagnosed HCC, 0.33% presented with brain metastases. Patients with bone or lung metastases had greater odds of having brain metastases at diagnosis. Second, HCCBM was such a fatal disease that median OS for the unique group was 2.40 months. Unmarried and bone metastases patients suffered significantly shorter survival time. Third, we constructed an easy-to-use nomogram model that might facilitate individualized prediction of OS in HCCBM patients.

Compared to other extrahepatic metastases sites, brain metastases from HCC is very rare [19]. In our study, the incidence of brain metastases from HCC was 0.33%. The frequency of HCCBM reported in other studies ranges from 0.2% to 2.2% [4, 20–23]. Yamakawa reported 0.9% of 1, 702 enrolled HCCs in the Shizuoka Cancer Center registry were diagnosed with brain metastases between January 2003 and December 2011 [24]. Choi also reported 0.9% of 6,919 HCCs treated at Yonsei University Health System had a diagnosis of brain metastases between 1995 and 2006. However, less than 0.07% patients (N = 5) had brain involvement as their initial presentation [4]. Our figure was lower mainly due to no metastatic cases could develop brain metastases in their following disease course.

We analyzed risk factors for the development of brain metastases from HCC using multivariable logistic regression. It revealed that bone metastases and lung metastases were associated with significantly greater odds of having brain metastases at diagnosis. Choi summarized that 43 of the 62 HCCBM patients had lung involvement [4]. Natsuizaka reported that all 5 HCCBM patients had lung lesions [25]. Seinfeld suggested that brain metastases from HCC could be secondary to lung deposits [20]. Hammond recommend that patients with lung metastases particularly undergo neuroimaging at time of diagnosis, even in the absence of symptoms [12]. Notably, our data considered the surveillance neuroimaging might necessary for both bone and lung metastases patients.

HCCBM generally is a late and detrimental stage with short expected survival after diagnosis. HCCBM showed a median survival of 2.40 months in the present study. Choi reported an overall median survival of 6.8 weeks in 62 Korean patients [4]. Chang reported the median survival of was 1 month after reviewed 45 Taiwanese patients [5]. Surgery [4, 5, 26], radiotherapy [4, 5, 26], and Gamma Knife [27] were reported to have positive effects on survival. However brain metastases frequently develops in patients with advanced stage, palliative therapy tends to be selected. Only one in our final 85 survival study patients underwent surgery. Additionally, radiotherapy data was no longer available in SEER. Thus, age, sex, race, marital status, insurance status, bone metastases, and lung metastases entered the Cox model, although treatment modality showed significant for survival in the previous study. Married status and absence of bone metastases showed favorable OS in multivariate analysis. Further studies regarding significant prognostic factors for survival in HCCBM are warranted.

This study is the first to construct a nomogram in predicting the clinical outcome of HCCBM. However, there are several acknowledged limitations to this study. First, the possible error resulted from limited patients (N = 85) could not be neglected. Therefore, we applied an internal validation with a resampling method by bootstrapping the entire cohort to calibrate the nomogram. Second, the SEER database only collected information about the disease at the first diagnosis, and patients might subsequently develop brain metastases later in their disease course. Third, bias from the treatment was not recorded in the SEER database. Finally, information about etiology (hepatitis viral infections and alcohol consumption), the number of metastases, body mass index, performance status, comorbidities is not available in the SEER database.

## Conclusion

Our study provided insight into the epidemiology of HCCBM patients. Our user-friendly nomogram may offer prognostic assessment for individual HCCBM. Further prospective clinical trials study will be warranted to validate our results.

## Supporting information

S1 Data Patients data. This is the all included patients’ information.
(XLSX)
